# A Specialized Clinical Laboratory Center for the Coronavirus Disease 2019 (COVID-19) in Wuhan Leishenshan Hospital During the COVID-19 Outbreak

**DOI:** 10.1017/dmp.2020.293

**Published:** 2020-08-12

**Authors:** Yaofei Xie, Wenwen Wu, Wen Xie, Yi Jin, Xiaodong Tan

**Affiliations:** School of Health Sciences, Wuhan University, Wuhan, Hubei, China; School of Public Health and Management, Hubei University of Medicine, Wuhan, Hubei, China; Department of Laboratory Medicine, Zhongnan Hospital of Wuhan University, Wuhan, China; Department of Laboratory Medicine, Leishenshan Hospital, Wuhan, Hubei, China; School of Nursing, Wuchang University of Technology, Wuhan, Hubei, China

**Keywords:** COVID-19, emergency, hospital, laboratory, test

## Abstract

Responding to the extreme scarcity of medical resources during the early outbreak of the coronavirus disease (COVID-19) in Wuhan, China, an emergency specialist hospital of Leishenshan started to construct on January 26, 2020, and accommodate patients on February 6, 2020. The clinical laboratory center of Leishenshan Hospital (CLCLH) was constructed at the same time within 11 days to support the treatment of inpatients in Leishenshan Hospital and the testing of suspected patients from different fever clinics in Wuhan. The CLCLH could perform a total of 320 clinic, 299 biochemistry, 31 microorganism, and 47 infection and immunity examinations per day. It could also complete an average of 239 nucleic acid tests and 118 severe acute respiratory syndrome coronavirus 2 (SARS-CoV-2) antibody examinations per day. No suspected cases were documented among the health care workers during the operation of the CLCLH. The construction and operation experiences of the CLCLH is provided in this study and might be used by other countries as reference. The content of this study is divided into 4 parts: (1) the establishment of the CLCLH, including its layout and medical resource allocation; (2) the major testing items; (3) the specific procedure of COVID-19 indicator examination; and (4) the standardized personal protection measures.

The coronavirus disease (COVID-19) pandemic outbreak is a global public health and safety emergency. Large-scale testing is key to achieving “early test, early diagnosis, early treatment, and early isolation.”^1,2^ Therefore, taking advantage of the second window of opportunity and quickly and effectively isolating confirmed cases are significant to cut off the potential transmission in countries that have adopted varied lockdown measures.^3,4^ However, the test scale remains limited for countries with increasingly severe pandemic situations because the effect of COVID-19 directly results in the collapse of the medical system and the medical test system. Thus, the test procedures must be improved scientifically to address the current health emergency.

As the epicenter of the COVID-19 pandemic outbreak, the emergence of massive cases has posed a great challenge to the existing medical resources in Wuhan. The Leishenshan Hospital with 1600 beds and 32 wards began construction on January 26, 2020, to address this problem, and it started operation on February 6, 2020. Meanwhile, the clinical laboratory center was constructed to support the treatment of inpatients in the Leishenshan Hospital and scale up the COVID-19 testing in Wuhan.

The Leishenshan Hospital was closed on April 15, 2020. Only 5 new confirmed cases were documented in Wuhan from March 15, 2020, to April 15, 2020, thereby validating certain achievements of the COVID-19 prevention and control work. The clinical laboratory center of Leishenshan Hospital (CLCLH) played an important role in the process of Wuhan’s fight against COVID-19. The CLCLH provided adequate routine testing for inpatients and relieved the burden of existing medical testing systems. This study is a systematic exposition of the construction and operation experience of the CLCLH. The main objective of this study is to provide a reference for epidemic prevention and control in other countries.

## ESTABLISHMENT OF THE CLCLH

Leishenshan Hospital is 1 of the 2 emergency specialty hospitals in Wuhan in response to the COVID-19 outbreak regarding Beijing’s Xiaotangshan Hospital. The CLCLH is an important part of Leishenshan Hospital. It was built in parallel with the hospital ward in 11 days and officially started its operations on February 15, 2020.

### Sources of Necessary Resources

The rapid establishment and operation of the CLCLH are based on adequate resource support. The government acknowledged their responsibility and provided funds to the infrastructure construction, including power supply, communication, and water supply. Most of the equipment was donated by the manufacturer through the Red Cross. The protective materials were uniformly allocated by the government due to its shortage in the early stages of the COVID-19 outbreak. Finally, the testing reagents and consumables were purchased by the hospital using the funds provided by the government.

### Main Construction Purposes of the CLCLH

The CLCLH has 2 major responsibilities: (1) provide testing for the treatment of COVID-19 inpatients in Leishenshan Hospital and (2) support large-scale testing of suspected patients from different fever clinics in Wuhan.

### Layout of the CLCLH

The biosecurity rating of the CLCLH is Level 3, based on the standards established by the US Centers for Disease Control and Prevention and the National Institutes of Health. Supplemental Figure 1 displays the layout of the CLCLH. This infrastructure is divided into 2 areas according to the function of each room. The core of the CLCLH is a negative pressure test chamber with a total area of 342.2 m^2^, which includes a negative pressure testing room (Supplemental Figure 2) and a sample sorting room. The latter is used by dedicated staff to sort out the received samples before the inspection. This center also includes an auxiliary functional area where the buffer room, the protective clothing changing room, the sample receipt room, and the pollutant packing room are located.

### Staff Composition

The working team of this center was selected by the Leading Group for Novel Coronavirus Prevention and Control from other hospitals in Wuhan. The team was composed of 34 medical staff, 4 of whom have advanced professional titles and 3 have polymerase chain reaction (PCR) qualification certificates. Most of them were technicians. The principal was responsible for quality and safety management.

### Equipment

The CLCLH is equipped with 24 kinds of instruments and devices. The specific names and quantities are shown in Supplemental Table 1.

## TESTING ITEMS

The testing items of the CLCLH are divided into 2 categories. The first category refers to the routine examinations of inpatients, including clinical, biochemical, microbiological, and infection and immune examinations. The second category refers to the indicators of severe acute respiratory syndrome coronavirus 2 (SARS-CoV-2) infection, including etiological and serological examinations. The main item of the etiological indicator is SARS-CoV-2 nucleic acid detection. Serological examinations mainly contain the SARS-CoV-2 antibody, the SARS-CoV-2-specific immunoglobulin M (IgM) antibody, and the total antibody. These samples were collected from inpatients in Leishenshan Hospital and other fever clinics in Wuhan.

**FIGURE 1:**
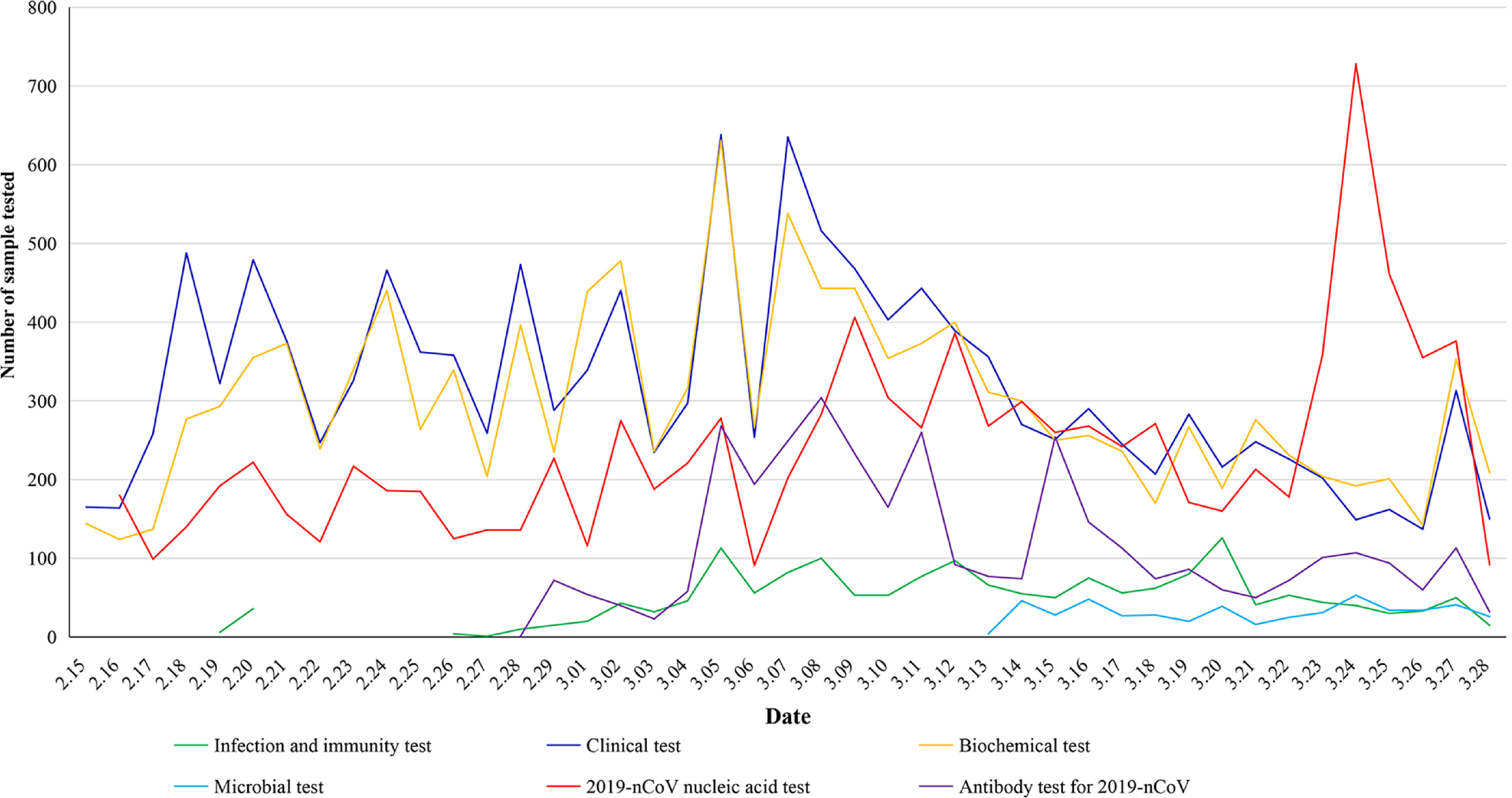
Workload Statistics of the Clinical Laboratory Center of Leishenshan Hospital.

The workload statistics are shown in Figure 1. The comprehensive testing programs can meet the needs of patients for diagnosis and treatment. The CLCLH completed analysis of a total of 42 369 samples from February 15, 2020, to March 28, 2020, including 10 038 SARS-CoV-2 nucleic acids tests and 3526 SARS-CoV-2 antibody examinations.

## PROCEDURE OF THE COVID-19 INDICATOR EXAMINATION

All samples must undergo a strict procedure from entering the laboratory center to the completion of the test, especially the disinfection work: (1) All samples are collected and placed in the sample transfer boxes, and the boxes need to pass through the transfer bin (Supplemental Figure 3) to enter the negative pressure testing room. Sample transfer boxes are disinfected during the transfer process; (2) Testers will then check the integrity of all the samples. The transfer boxes are disinfected again and transferred out from the cleaning bin; (3) All of the sample packing are disinfected. The disinfected samples are then divided into 3 categories: body fluid samples, centrifuge free samples, and samples to be centrifuged; (4) Body fluid samples and centrifuge-free samples are accepted after scanning codes. The remaining samples are centrifuged as needed. The centrifuge is left open and disinfected 10 minutes after centrifugation; (5) The biosafety cabinet is sterilized with medical alcohol before sample entering; (6) After testing, all samples should be placed in the safety cabinet to be sterilized by ultraviolet for 30 minutes; (7) The waste is disposed, and the biosafety cabinet is sterilized with medical alcohol; (8) Finally, the waste materials and samples are packaged and transported.

## PRECAUTIONS OF PERSONAL PROTECTION

The samples received by the laboratory center are all suspected samples, which can easily produce aerosols through centrifugation, cover opening, blowing, and other experimental operations. Considering that aerosol transmission is one of the important transmission routes of COVID-19,^5^ the testers need to take standardized personal protective measures.

### Basic Principles

All opening operations must be conducted in biosafety cabinets. All used testing and auxiliary equipment must be wiped clean with 75% alcohol and sterilized by spraying 5% 84-disinfectant. Centrifuges are high-risk equipment that can cause aerosol pollution, and thus they need to be soaked for disinfection.

### Procedure for the Testers

Safety measures include the following:

1. Wash the hands before the experiment, and then wear hats, goggles, masks (KN95, N95 and other specifications with filtering efficiency of more than 95%), latex gloves (2 layers of different colors), protective clothing (preferably conjoined to prevent the inner layer of clothing from being contaminated), and shoe covers.
2. If pollution is observed during the experiment, the protective equipment must be replaced immediately. Even if no obvious pollution exists, the protective equipment must still be changed every 4 hours.
3. Disinfect the hands and replace the gloves before taking it out from the biosafety cabinet.
4. After taking off protective clothing, do not touch the face (including mouth, nose, and eyes), personal belongings, or work items in a clean area before washing hands.
5. All handwashing processes must be in strict accordance with the requirements of the 7-step handwashing method. After washing the hands, rub them with 75% alcohol for 1–3 minutes.

## DISCUSSION

The Leishenshan Hospital is a 1600-bed, triple-A medical facility built for the treatment of COVID-19 in Wuhan. It took just 11 days from construction decision to accepting patients. As a crucial component of Leishenshan Hospital, the CLCLH was built following 3 principles: rapid construction, biosafety, and efficiency. The construction of the large-area negative pressure bin is the key.

The layout of the CLCLH is the foundation of biosafety and efficiency. It was designed according to the sample testing process. From the sample receiving to the final sample processing, each operation step has its space partition. This structure guarantees the biosafety and results in an orderly operation. The CLCLH ensures efficient operation through the configuration of testing equipment and personnel. In addition to the treatment of routine patients, the test needs of diagnosis and treatment of COVID-19 was considered, especially the tests of many suspected patients from the COVID-19 designated fever clinics, which were set up by the hospitals in Wuhan in response to the COVID-19 outbreak.

The CLCLH provided adequate routine testing items during the COVID-19 outbreak in Wuhan to support the diagnosis and treatment for inpatients. It also relieved the burden of existing medical testing systems by testing suspected patients from the COVID-19 designated fever clinics in Wuhan. Given the medical protection material application and strictly implemented testing procedures, especially disinfection operations, no suspected cases were documented among the testers during the operation of the CLCLH.

Thus, as a component of the emergency medical system, the rapidly built specialized clinical laboratory center for the treatment of COVID-19 is an effective practice. Considering laboratory testing is not only the basis of patients’ treatment, but also the core of specific control measures, other countries and communities worldwide might boost their response to the current COVID-19 pandemic by embracing such experience.

## Abbreviations

COVID-19: coronavirus disease
CLCLH: clinical laboratory center of Leishenshan Hospital
PCR: polymerase chain reaction
IgM: immunoglobulin M

## Supplementary material

For supplementary material accompanying this paper visit https://doi.org/10.1017/dmp.2020.293. click here to view supplementary material
