# Supplementary material for: A Specialized Clinical Laboratory Center for the Coronavirus Disease 2019 (COVID-19) in Wuhan Leishenshan Hospital During the COVID-19 Outbreak
Source: Disaster Med Public Health Prep. 2020 Aug 12:1–4. doi: 10.1017/dmp.2020.293 (PMC7588718; doi:10.1017/dmp.2020.293)
Supplement: Supplementary file 1 [file S1935789320002931sup.zip › S1935789320002931sup001.docx]

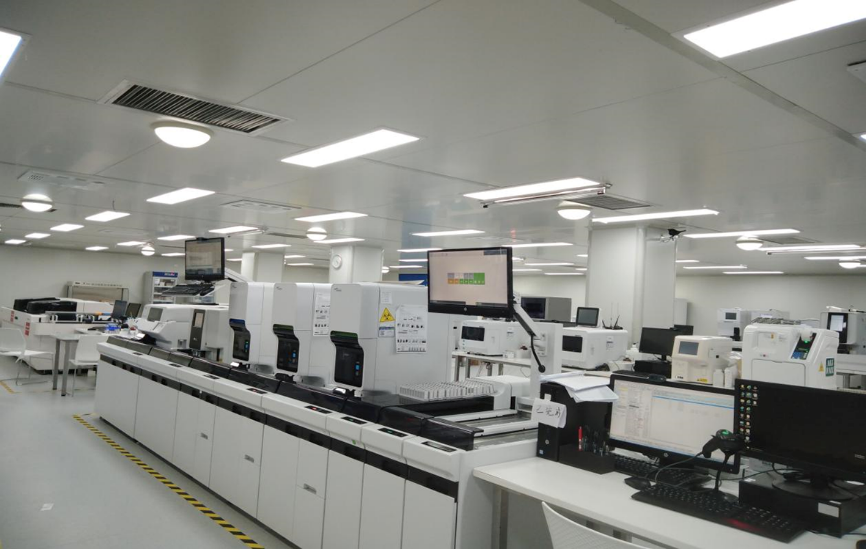


**Supplemental Figure 2. Equipment of negative pressure testing room of the clinical laboratory center of Leishenshan Hospital**
