# Supplementary material for: A Specialized Clinical Laboratory Center for the Coronavirus Disease 2019 (COVID-19) in Wuhan Leishenshan Hospital During the COVID-19 Outbreak
Source: Disaster Med Public Health Prep. 2020 Aug 12:1–4. doi: 10.1017/dmp.2020.293 (PMC7588718; doi:10.1017/dmp.2020.293)
Supplement: Supplementary file 1 [file S1935789320002931sup.zip › S1935789320002931sup002.docx]

**Supplemental Table 1. Equipment of the clinical laboratory center of Leishenshan Hospital**

| **Equipment** | **Number** |
| --- | --- |
| Automatic biochemical analyzer | 2 |
| Automatic chemiluminescence analyzer | 1 |
| Automatic blood analyzer | 7 |
| Specific protein analyzer | 2 |
| Automatic urine analyzer | 1 |
| Automatic coagulation detector | 1 |
| Low-speed normal temperature centrifuge | 4 |
| Uninterruptible power system | 1 |
| Ultrapure water machine | 1 |
| Automatic blood sedimentation analyzer | 1 |
| Autoclave | 2 |
| Special centrifuge for blood type serum | 2 |
| Plasma thawing apparatus | 1 |
| Pipetting gun | 50 |
| Microscope | 3 |
| Blood gas analyzer | 30 |
| Automatic blood type analyzer | 1 |
| Platelet shock storage box | 2 |
| Blood bank refrigerator | 2 |
| Low-temperature refrigerator | 2 |
| Ultra-low temperature refrigerator | 1 |
| Medical refrigerator | 8 |
| Class [Ⅱ](http://xueshu.baidu.com/usercenter/paper/show?paperid=4f715c7e182be404efbe9eba2d7709ad&site=xueshu_se)biosafety cabinet | 2 |
| Blood cryogenic operation table | 2 |
| Nucleic acid isolation machine | 3 |
